# Associations of self- and informant-reported functional impairment with cognitive performance and incident dementia

**DOI:** 10.1016/j.tjpad.2026.100482

**Published:** 2026-01-17

**Authors:** Yaqing Gao, Paola Zaninotto, Andrew Steptoe

**Affiliations:** a Department of Epidemiology and Public Health, Institute of Epidemiology and Health Care, University College London, London, UK; b Department of Behavioural Science and Health, Institute of Epidemiology and Health Care, University College London, London, UK

**Keywords:** Functional impairment, Activities of daily living, Instrumental activities of daily living, Cognitive function, Executive function, Memory, Dementia

## Abstract

**Introduction:**

Functional impairment is central to dementia diagnosis, typically assessed through basic and instrumental activities of daily living (ADLs/IADLs) reported by participants or informants. However, it remains unclear which items and reporting sources best capture cognition-related functional impairment.

**Methods:**

We analysed data from 1,050 adults aged ≥65 years in the English Longitudinal Study of Ageing Harmonised Cognitive Assessment Protocol. Self- and informant-reported ADL and IADL impairments were examined at overall and item levels. Associations with general and domain-specific cognition were estimated using linear regression, and with incident dementia over 6.7 years using Cox models, both adjusted for socioeconomic, lifestyle, and health factors.

**Results:**

ADL and IADL impairment proportions were similar across self- and informant-reports, with modest concordance. Informant-reported, but not self-reported, impairments were consistently associated with poorer cognition across domains, particularly executive function (informant-reported ADL: β = –0.71 SD, 95% CI –0.89 to –0.53) and memory (informant-reported IADL: β = –0.46 SD, 95% CI –0.60 to –0.32), and higher dementia risk (ADL: HR = 3.13, 95% CI 1.23 to 7.96; IADL: HR = 5.00, 95% CI 2.40 to 10.43). The strongest associations were observed for managing money and tasks involving episodic or visuospatial memory, especially among individuals with intermediate education and when informants had higher education or daily contact.

**Discussion:**

Informant-reported IADLs, particularly those involving financial management and memory, may be strong indicators of cognitive impairment and dementia risk. Emphasising these items and informant characteristics may improve population surveillance of dementia and inform outcome selection in preclinical dementia trials targeting early functional decline. These findings should be interpreted in the context of a population-based sample with attrition.

## Highlights

• ADL and IADL impairment proportions were similar across self- and informant-reports, but concordance between them was modest.
• Self-reported measures of functional impairment appeared limited in capturing poor cognition or dementia risk, whereas informant-reports showed stronger and more consistent associations.
• The strongest associations were observed for informant-reported IADLs involving episodic and visuospatial memory.
• Associations were independent of socioeconomic, lifestyle, and health factors.
• Associations were stronger among individuals with intermediate education and when informants were highly educated or in daily contact.

## 1 Introduction

Accurate ascertainment of dementia in large, representative population studies is essential for tracking trends, assessing disparities, and examining risk factors. Functional impairment is central to dementia diagnosis, which requires cognitive decline severe enough to interfere with everyday activities;[1] and its progression—from subtle difficulties in complex instrumental tasks to loss of independence in basic activities—forms the basis of clinical staging from mild cognitive impairment (MCI) to severe dementia[2] Accordingly, loss of functional capacity is embedded in algorithmic dementia/MCI classification systems used in large-scale cohort studies worldwide[3,4] Improving the identification of functional measures that are potentially sensitive and specific to early cognitive decline could enable scalable screening, accurate risk stratification, and improved estimates of dementia burden to guide resource allocation in ageing populations.

Functional ability in older adults is commonly assessed through activities of daily living (ADLs), which capture basic physical skills such as dressing, toileting, getting in and out of bed, and eating, and instrumental activities of daily living (IADLs), which reflect more complex tasks required for independent living, such as managing finances and medications[5,6] ADLs are mastered early in life, highly routinised, and often remain intact until later disease stages,[7] whereas IADLs are more cognitively demanding and typically decline earlier[8] Beyond cognition, limitations with ADL and IADL have been associated with physical health,[9] mental health,[10] education,[11] personal wealth,[12] and area-level deprivation[13] However, most research examining links between ADL/IADL impairment and cognition has not simultaneously accounted for these factors, leaving it unclear whether observed associations reflect underlying cognitive impairment or comorbid health and social disadvantage.

A further question is whether to rely on self- or informant-reported functional abilities. Some studies suggest that individuals overestimate their functional impairments compared with informant reports,[14] whereas others indicate that people with poorer cognition may underreport impairments due to reduced awareness or insight[15,16] Links between self-reported functional impairment and objective cognition or dementia biomarkers (e.g., reduced grey matter volume, elevated p-tau in cerebrospinal fluid, and amyloid deposition) have been inconsistent,[[17], [18], [19], [20]] whereas informant-reported impairment has shown more consistent associations[[20], [21], [22]] To our knowledge, only two studies have conducted direct within-study comparisons of self- and informant-reported measures,[16,20] both based on clinic-based samples, highlighting the need for more comprehensive evidence from population-based studies. Moreover, reporting may vary with participant factors such as age and education,[23] or with informant characteristics such as education and contact frequency,[24] yet few studies have examined whether associations differ across these groups.

At the population level, functional impairment is typically defined dichotomously (e.g., difficulty with one or more ADLs or IADLs)[12,25] and severity expressed through summary scores across multiple activities[26] Such aggregate measures do not specify which activities are affected, meaning individuals with very different patterns of impairment may fall into the same category, masking item-level differences. Meanwhile, most studies have examined global rather than domain-specific cognition, despite evidence that particular functional items may be more closely linked to specific domains[27,28]—an important consideration for dementia classification algorithms, which require evidence of impairment in one or more domains[1] Few studies have directly examined the relationship between functional impairment and incident dementia, and those that did often relied on follow-up cognitive batteries or clinical assessments to ascertain dementia [16,29] This approach is prone to attrition bias, as participants who remain in studies tend to have better cognition than those who drop out, potentially underestimating associations [30]

This study used data from the English Longitudinal Study of Ageing Harmonised Cognitive Assessment Protocol (ELSA-HCAP),[31] a population-based study of dementia and cognitive ageing that includes both respondent and informant interviews. Respondents completed an extensive cognitive battery covering multiple domains and reported on their own ADL and IADL abilities, while informants provided parallel ratings of functional status. We compared self- and informant-reported ADL and IADL impairments—both overall and at the item level—in relation to domain-specific and general cognitive factor scores and to incident dementia, adjusting for sociodemographic, lifestyle, and health factors, and examined whether associations differed by participant or informant characteristics.

## 2 Methods

### 2.1 Study design

ELSA-HCAP is a sub-study of the English Longitudinal Study of Ageing (ELSA). ELSA is a nationally representative longitudinal panel of adults aged ≥50 years in England, established in 2002 and re-interviewing participants every two years through 2024 (wave 11)[32] The initial sample (>11,000) and periodic refreshment samples were added to maintain representativeness of the English population aged ≥50 years. Data are collected through computer-assisted personal interviews and self-completion questionnaires covering health, social, economic, and wellbeing domains.

The first ELSA-HCAP was conducted in 2018[31] Eligible participants were ELSA core members aged ≥65 years who had completed at least one respondent interview (rather than informant-only) at wave 7 (2014–2015) or wave 8 (2016–2017). Individuals in care or nursing homes were also eligible if they had originally joined ELSA from the community and could provide consent. Participants with dementia or lower cognitive function were oversampled.

The HCAP comprised paired in-person interviews: one with the respondent and one with an informant nominated by the respondent who was familiar with their health and daily functioning. The respondent interview included a detailed neuropsychological battery covering multiple cognitive domains and questions on health and everyday function. The informant completed a self-administered questionnaire on the respondent’s functional abilities.

Of 1,684 eligible participants, 1,273 completed the interview (response rate 75.6%)[31] A total of 1,050 informant interviews were obtained (82.5% of eligible participants). This study included all individuals with an informant interview (N = 1,050).

### 2.2 Functional impairments

HCAP assessed self-reported functional abilities using items from activities of daily living (ADLs) and instrumental activities of daily living (IADLs). Participants were asked whether they had difficulty (yes/no) performing six ADLs (bathing, dressing, eating, toileting, getting in or out of bed, and walking across a room) and nine IADLs (performing household tasks, managing money, using a map, recognising danger, preparing meals, shopping, making telephone calls, communicating, and taking medications). Self-reported ADL impairment was defined as difficulty with ≥1 ADL, and self-reported IADL impairment as difficulty with ≥1 IADL, consistent with criteria for functional impairment widely used in prior studies,[[10], [11], [12]] including those incorporated into dementia classification[4]

Informant-reported functional impairment was measured using the Blessed Dementia Rating Scale,[33] Parts 1 (IADLs) and 2 (ADLs). Part 2 assessed three ADLs (eating, toileting, dressing), rated from no impairment to minor, major, or complete dependence. For item-level analyses, responses were dichotomised as no impairment versus impairment (minor, major, or complete dependence). Part 1 covered eight IADLs (performing household tasks, coping with money, remembering short lists, finding one’s way indoors, navigating familiar streets, grasping situations or explanations, recalling recent events, and dwelling in the past), each rated as no loss, some loss, or severe loss (dwelling in the past: none of the time, sometimes, frequently). For item-level analyses, responses were dichotomised as no impairment (no loss) versus impairment (some loss or severe loss). For overall measures, informant-reported ADL impairment was defined as difficulty with one or more ADLs in Part 2. For IADLs, Part 1 responses were scored as no loss = 0, some loss = 0.5, severe loss = 1 (range 0–8). Informant-reported IADL impairment was defined as a total score ≥2, a cut-off consistently used in dementia classification algorithms across HCAP studies in the US, India, and multiple European countries[3,4,34] In sensitivity analyses, we applied a less conservative threshold of ≥1.5 to define informant-reported IADL impairment.

### 2.3 Cognitive performance

The HCAP battery included a comprehensive set of cognitive tests designed to capture domains most affected by cognitive ageing. Based on established neuropsychological theory and confirmatory factor analyses,[35] items were grouped into five domains: orientation, executive function, language, memory, and visuospatial ability. Some tests, such as the Mini-Mental State Examination, contributed multiple items spanning different domains. Test names, domains, descriptions, and item-level missingness are shown in Table S1.

We used previously derived factor scores summarizing individual tests into domain-specific and general cognition measures[36] Missing item responses were imputed using a block-sequential and chained approach, drawing on extensive longitudinal covariates from ELSA and ELSA-HCAP. Imputed values were generated as pseudo-random draws from estimated conditional distributions. Factor scores were estimated using the Expected a Posteriori method, with comparisons against Bayesian plausible values to assess precision. To facilitate interpretation and comparison, scores were standardized (mean = 0, variance = 1), and their distributions are shown in Fig. S1.

### 2.4 Incident dementia

Incident dementia was defined as the first record of a dementia diagnosis from three sources: (i) self-reported physician diagnosis of Alzheimer’s disease or dementia across all ELSA core and HCAP waves, using the interview date as the proxy date of diagnosis; (ii) linked Hospital Episode Statistics (HES) inpatient and outpatient records, covering all National Health Service hospital admissions in England; HES inpatient data identify all-cause dementia with 78% sensitivity and 92% specificity;[37] and (iii) national mortality registers. Both HES and mortality registers used International Classification of Diseases codes. We applied the dementia code list developed by the UK Biobank Outcome Adjudication Group, which has demonstrated good validity, with a positive predictive value of 84.5% (Table S2)[38,39]

### 2.5 Covariates

To reduce misclassification, we used the most recent measures available, prioritizing the HCAP questionnaire and otherwise drawing from the latest ELSA core interview (wave 7 or 8). Tables S2–S3 detail variable definitions, categorizations, and the source of each assessment.

Demographic covariates included age at HCAP participation (years) and sex (male/female). Socioeconomic covariates included living alone (yes/no), educational attainment (<O level, O level, ≥A level), quintiles of total net family wealth, and quintiles of the Index of Multiple Deprivation, a small-area measure (∼1,500 residents) combining income, employment, education, health, crime, barriers to housing and services, and living environment[40] Lifestyle covariates were smoking status (past/current vs. never) and alcohol consumption (≥5 vs. <5 days/week). Health covariates captured history before HCAP of diabetes, hypertension, cardiovascular disease (angina, myocardial infarction, heart failure, stroke), chronic lung disease, and arthritis, identified through self-reports in HCAP or prior ELSA waves and linked HES records. Depressive symptoms were assessed using the 8-item Center for Epidemiologic Studies Depression Scale (CESD-8), with endorsement of ≥4 negative items indicating likely depression[41]

For analyses of informant-reported measures, additional covariates included informant sex (male/female), educational attainment (<O level, O level, ≥A level), and frequency of contact with the respondent (daily vs. less than daily).

### 2.6 Statistical analysis

We first described the proportion of participants reporting difficulty with each ADL and IADL item, as well as the distribution of summed impairment scores, using histograms. Baseline sociodemographic and health characteristics were summarised for the total sample and stratified by the presence of self- or informant-reported ADL/IADL impairment. Categorical variables were reported as counts and percentages, and continuous variables as means with standard deviations. Pearson correlations were estimated between self- and informant-reported ADL and IADL, at both overall and item levels.

We used linear regression to examine associations between self- or informant-reported impairment (individual items and overall ADL/IADL impairment) and cognitive performance, assessed by factor scores for general cognition, executive function, memory, and language; orientation and visuospatial scores were excluded due to non-normal distributions (Fig. S1). To assess the extent to which these associations were independent of potential confounders, covariates were added sequentially: Model 1 adjusted for age and sex; Model 2 additionally adjusted for socioeconomic and lifestyle factors (and for informant characteristics when the exposure was informant-reported); and Model 3 further adjusted for health-related variables, which served as the fully adjusted primary model. As the proportion of missing data was low (<5% for most exposures and covariates; Table S4), complete-case analyses were conducted.

Associations between functional impairment (individual items and overall ADL/IADL impairment) and incident dementia were assessed using Cox proportional hazards regression, with time since baseline as the timescale. Participants were followed from their baseline HCAP assessment until dementia diagnosis, death, or the latest available interview date in ELSA or HCAP (15 Oct 2024), whichever came first. Models were sequentially adjusted using the same covariate sets as described above to evaluate robustness of associations to increasing levels of adjustment.

Effect modification of the association between ADL/IADL impairment and general cognitive performance was tested by participant age (<70, 70–80, >80 years), sex, and education for self-reported measures, and additionally by informant sex, education, and contact frequency for informant-reported measures. Analyses were stratified by subgroups, with general cognitive factor scores re-standardised within each subgroup for comparability. Interactions were evaluated using Wald F-tests.

All analyses were weighted to account for differential sampling and participation probabilities in ELSA-HCAP.

## 3 Results

**Fig. 1:**
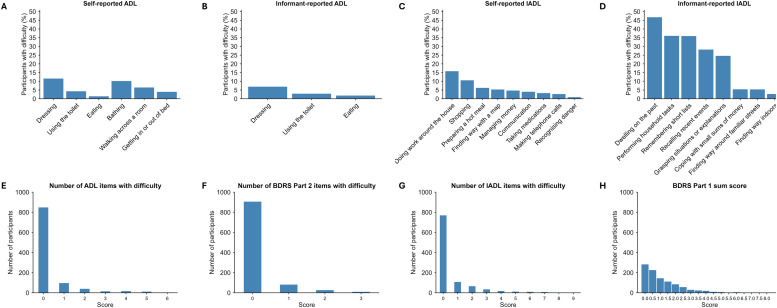
Proportion and distribution of self- and informant-reported functional impairments.

Across both self- and informant-reports, impairment with ADLs were less common than with IADLs (Fig. 1). For ADLs, dressing was the most frequently reported difficulty (11.7% self-reported, 7.1% informant-reported), followed by bathing (10.3% self-reported), while eating and toileting were reported by fewer than 5% of participants. Overall, 18.7% had self-reported ADL impairment and 8.8% had informant-reported ADL impairment. For IADLs, comparable items showed similar proportions between self- and informant-reports. For example, managing money (self-reported) versus coping with small sums of money (informant-reported) (4.8% vs 5.6%), and finding the way with a map (self-reported) versus finding the way around familiar streets (informant-reported) (5.4% vs 5.5%). Several informant-only items showed markedly higher proportions, including dwelling on the past (46.9%), and reporting difficulty in performing household tasks (36.2%) and remembering short lists (36.1%). By contrast, impairment in self-report-only items were less frequent, most commonly involving doing work around the house (16.0%) and shopping (10.7%). Despite these item-level differences, the overall proportion of IADL impairment was similar between self- and informant-reports (23.5% vs 19.8%). Using a less conservative cutoff (BDRS Part 1 ≥1.5 instead of ≥2) for informant-reported IADL impairment increased the proportion classified as impaired to 29.6% (Table S5).

ADL, activities of daily living; IADL, instrumental activities of daily living; BDRS, Blessed Dementia Rating Scale. Panels A–D show the proportion of participants reporting difficulty with each item: (A) self-reported ADLs, (B) informant-reported ADLs (based on items in BDRS Part 2), (C) self-reported IADLs, and (D) informant-reported IADLs (based on items in BDRS Part 1). Panels E–H show distributions of composite scores: (E) number of self-reported ADL items with difficulty, (F) number of informant-reported ADL (BDRS Part 2) items with difficulty, (G) number of self-reported IADL items with difficulty, and (H) BDRS Part 1 (used to measure informant-reported IADL) sum score. All estimated proportions were weighted to account for differential sampling and participation probabilities.

Self-reported ADL and IADL impairments were strongly correlated (r=0.56; Fig. S2), whereas informant-reported measures showed a lower correlation (r=0.33). Furthermore, correlation coefficients between self- and informant-reported measures were low, indicating weak concordance. At the item level, the highest ADL agreement was for dressing (r=0.36), while eating and toileting showed low to moderate correlations (r=0.1–0.3). For IADLs, the strongest correlations were between self-reported managing money and informant-reported coping with small sums of money (r=0.54), and between doing work around the house and performing household tasks (r=0.40). Most other IADL items showed low to moderate correlations.

Compared with the overall sample, participants with functional impairments (either self-reported or informant-reported) were generally older and more likely to live alone (Table 1). Lower education, reduced individual wealth, and greater area-level deprivation were evident across all impairment groups, particularly among those with self-reported impairments. All impairment groups also had higher levels of risk factors and chronic conditions: informant-reported ADL impairment showed the highest proportions of past/current smoking and histories of diabetes, hypertension, chronic lung disease, and cardiovascular disease, whereas self-reported ADL impairment showed the highest proportions of arthritis and depressive symptoms (33.5%).

**Table 1:** Baseline characteristics of participants overall and by presence of self- and informant-reported ADL and IADL impairment.

Characteristic	Overall	ADL impairment		IADL impairment	
		Self-reported a	Informant-reported b	Self-reported c	Informant-reported d
N	1050 (100.0)	197 (18.8)	130 (12.5)	276 (26.3)	273 (26.1)
Age, mean (SD)	75.4 (7.2)	78.8 (8.1)	80.7 (7.7)	79.4 (8.0)	79.3 (7.6)
Female, n (%)	569 (52.9)	116 (59.0)	66 (51.2)	171 (62.3)	149 (57.7)
Living alone, n (%)	274 (24.3)	71 (31.2)	38 (28.1)	102 (34.9)	90 (33.4)
Education					
<O-level	459 (42.5)	114 (57.0)	75 (58.0)	158 (56.0)	146 (54.0)
O-level	267 (24.8)	41 (19.1)	26 (19.0)	61 (20.5)	55 (17.3)
≥A-level	323 (32.7)	41 (23.9)	28 (23.0)	56 (23.5)	71 (28.7)
Wealth quintile e, n (%)					
Quintile 1 (Lowest wealth)	192 (17.8)	52 (26.4)	37 (32.6)	67 (26.8)	69 (25.5)
Quintile 2	208 (20.1)	49 (26.5)	31 (22.1)	63 (23.8)	69 (27.8)
Quintile 3	209 (21.3)	38 (20.2)	22 (14.7)	56 (20.0)	52 (21.6)
Quintile 4	204 (20.5)	33 (17.4)	23 (20.6)	48 (18.1)	42 (14.3)
Quintile 5 (Highest wealth)	203 (20.3)	17 (9.4)	13 (9.9)	31 (11.3)	33 (10.8)
IMD quintile f, n (%)					
Quintile 1 (Least deprived)	227 (21.6)	28 (12.1)	22 (15.8)	42 (13.4)	52 (18.0)
Quintile 2	269 (24.8)	53 (21.4)	40 (27.5)	76 (23.9)	72 (23.6)
Quintile 3	206 (21.2)	36 (19.9)	20 (11.5)	53 (20.4)	51 (20.7)
Quintile 4	185 (18.0)	42 (24.6)	22 (22.1)	52 (21.2)	52 (24.4)
Quintile 5 (Most deprived)	138 (14.3)	34 (22.0)	22 (23.1)	46 (21.0)	38 (13.3)
Past/current smoking, n (%)	682 (65.0)	134 (67.1)	90 (71.0)	188 (69.8)	191 (69.9)
Drinking ≥5 days/week, n (%)	209 (22.6)	29 (17.4)	26 (21.9)	49 (20.9)	44 (16.6)
Diabetes, n (%)	196 (17.3)	41 (14.7)	29 (17.0)	60 (19.3)	61 (22.5)
Hypertension, n (%)	681 (62.4)	146 (73.3)	102 (78.0)	212 (77.9)	198 (69.9)
Chronic lung disease, n (%)	177 (15.8)	55 (25.3)	45 (39.2)	79 (27.1)	66 (22.7)
Arthritis, n (%)	606 (56.2)	157 (78.0)	93 (71.6)	209 (75.7)	183 (65.0)
Cardiovascular disease, n (%)	311 (24.8)	84 (39.0)	65 (52.7)	123 (41.5)	114 (41.4)
Depressive symptoms, n (%)	152 (13.5)	68 (33.5)	29 (22.7)	87 (31.2)	74 (28.5)

ADL, activities of daily living; IADL, instrumental activities of daily living; SD, standard deviation; IMD, Index of Multiple Deprivation. All estimated means, standard deviations, and proportions were weighted to account for differential sampling and participation probabilities.

a Defined as difficulty with ≥1 activity of daily living (ADL).

b Defined as difficulty with ≥1 ADL in Blessed Dementia Rating Scale (BDRS) Part 2.

c Defined as difficulty with ≥1 instrumental activity of daily living (IADL).

d Defined from BDRS Part 1, where responses were scored as no loss = 0, some loss = 0.5, severe loss = 1 (range 0–8); scores >2 indicated impairment.

e Net financial wealth measured at the benefit unit level (i.e., a single adult or a couple, plus any dependent children, sharing finances). Calculated as gross financial assets minus financial debt.

f Index of Multiple Deprivation: Area-level measure of socioeconomic deprivation based on participants’ postcode, incorporating income, employment, education, health, crime, housing, and environment.

**Fig. 2:**
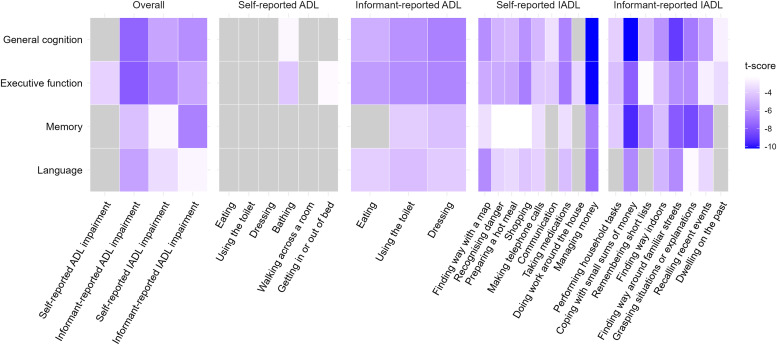
Associations between functional impairment and cognitive performance.

The final analytic sample comprised 1,050 participants with generally preserved cognition; among the 940 participants with complete Mini-Mental State Examination data, the 25th percentile score was 25, above the conventional threshold (<24) for cognitive impairment (Table S6). Overall, functional impairment measures were associated with lower cognitive performance across domains, except for self-reported ADL impairment, which showed only a weak association with executive function (Fig. 2). Adjustment for socioeconomic, lifestyle, and health factors attenuated estimates, but most associations remained statistically significant after full adjustment (Table S7). In the fully adjusted model, the strongest association was observed for informant-reported ADL impairment, corresponding to a 0.71 SD lower executive function score (β = –0.71, 95% CI –0.89 to –0.53) and a 0.66 SD lower general cognition score (β = –0.66, 95% CI –0.83 to –0.49). Informant-reported IADL impairment was also strongly associated with lower memory (β = –0.46, 95% CI –0.60 to –0.32) and general cognition (β = –0.36, 95% CI –0.48 to –0.25), and the association with general cognition weakened when the less conservative threshold of ≥1.5 for defining IADL impairment was applied (β = –0.28, 95% CI –0.39 to –0.18; Table S5).

ADL, activities of daily living; IADL, instrumental activities of daily living; Linear regression models tested associations of self- and informant-reported ADL/IADL impairment (overall and individual items) with factor scores for general cognition, executive function, memory, and language. Models were fully adjusted for demographics, socioeconomic, lifestyle, and health covariates and weighted to account for differential sampling and participation probabilities. P-values were FDR-corrected; grey indicates non-significant associations (FDR > 0.05), with darker blue denoting more negative t-scores (β/standard error).

At the item level, associations were consistently stronger for informant- than for self-reports (Fig. 2). Informant-reported ADLs (eating, using the toilet, and dressing) were consistently linked to lower scores across domains, particularly executive function, whereas most self-reported ADL items showed no significant associations. Informant-reported IADLs demonstrated the broadest and strongest associations, especially for coping with small sums of money and finding the way around familiar streets, with the largest differences observed in general cognition and memory. In contrast, self-reported IADLs showed more variable associations, with the strongest links observed for finding the way with a map, shopping, and managing money, particularly in relation to general cognition and executive function.

**Fig. 3:**
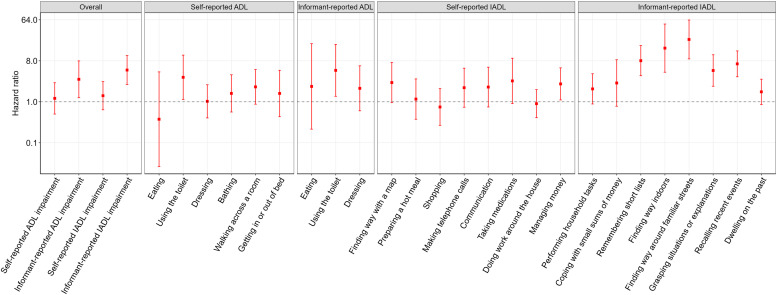
Associations between functional impairment and incident dementia.

ADL, activities of daily living; IADL, instrumental activities of daily living; Cox regression models tested associations of self- and informant-reported ADL/IADL impairment (overall and individual items) with incident dementia. Models were fully adjusted for demographics, socioeconomic, lifestyle, and health covariates and weighted to account for differential sampling and participation probabilities. Results are presented as hazard ratios with 95% confidence intervals.

Eighty-nine participants had a dementia diagnosis before HCAP; among the remaining 961, 101 developed dementia during follow-up of up to 6.7 years. Informant-reported IADL impairment was associated with more than a fivefold higher risk of dementia after full adjustment for socioeconomic, lifestyle, and health factors (HR 5.00, 95% CI 2.40 to 10.43; Fig. 3 and Table S8). When the less conservative cut-off of ≥1.5 was applied, the association was attenuated but remained strong (HR 3.82, 95% CI 1.90 to 7.67; Table S5). Informant-reported ADL impairment showed a similarly strong association (HR 3.13, 95% CI 1.23 to 7.96). Self-reported IADL impairment was linked to higher dementia risk in minimally adjusted models, but this was attenuated to the null after adjusting for sociodemographic and health factors, while self-reported ADL impairment showed no association in any model.

At the item level, both self- and informant-reported ADLs, as well as self-reported IADLs, showed weak and imprecise associations with dementia risk (Fig. 3). Informant-reported IADL items showed strong associations, with remembering short lists, finding ways indoors, finding the way around familiar streets, grasping situations or explanations, and recalling recent events each linked to more than a fourfold higher risk of dementia.

Significant subgroup differences were observed (FDR-adjusted p for interaction<0.05, Table 2). Associations between informant-reported IADL impairment and cognition were strongest among participants with intermediate (O-level) education, compared with those with either lower or higher qualifications, and were also stronger when informants had daily contact. Associations with informant-reported ADL impairment were stronger when informants had higher education.

**Table 2:** Effect modification of the associations between functional impairment and general cognitive performance.

Characteristic	Self-reported ADL impairment		Self-reported IADL impairment	
	β (95% CI)	*p* for interaction	β (95% CI)	*p* for interaction
Age				
<70	-0.29 (-0.74 to 0.15)	0.569	-0.37 (-0.74 to 0.00)	0.659
70–80	-0.21 (-0.40 to -0.02)		-0.49 (-0.68 to -0.30)	
>80	-0.11 (-0.36 to 0.14)		-0.38 (-0.61 to -0.14)	
Sex				
Male	-0.29 (-0.49 to -0.10)	0.278	-0.46 (-0.64 to -0.27)	0.250
Female	-0.05 (-0.21 to 0.12)		-0.23 (-0.38 to -0.08)	
Education				
<O-level	-0.23 (-0.43 to -0.03)	0.659	-0.38 (-0.57 to -0.19)	0.659
O-level	0.00 (-0.28 to 0.28)		-0.46 (-0.72 to -0.20)	
≥A-level	-0.09 (-0.33 to 0.16)		-0.22 (-0.45 to 0.01)	

	Informant-reported ADL impairment		Informant-reported IADL impairment	
	β (95% CI)	*p* for interaction	β (95% CI)	*p* for interaction
Age				
<70	-1.50 (-3.06 to 0.05)	0.690	-0.02 (-0.54 to 0.50)	0.250
70–80	-0.75 (-1.03 to -0.47)		-0.48 (-0.66 to -0.30)	
>80	-0.76 (-1.06 to -0.46)		-0.54 (-0.78 to -0.31)	
Sex				
Male	-0.60 (-0.86 to -0.35)	0.394	-0.43 (-0.66 to -0.20)	0.250
Female	-0.78 (-1.03 to -0.53)		-0.35 (-0.50 to -0.20)	
Education				
<O-level	-0.65 (-0.92 to -0.39)	0.362	-0.33 (-0.53 to -0.13)	0.006
O-level	-1.18 (-1.57 to -0.78)		-1.14 (-1.41 to -0.88)	
≥A-level	-0.54 (-0.89 to -0.20)		-0.34 (-0.54 to -0.14)	
Informant sex				
Male	-0.61 (-0.95 to -0.27)	0.138	-0.43 (-0.66 to -0.20)	0.659
Female	-0.75 (-0.96 to -0.54)		-0.35 (-0.50 to -0.20)	
Informant education				
<O-level	-0.41 (-0.73 to -0.09)	0.006	-0.64 (-0.87 to -0.41)	0.250
O-level	-0.57 (-0.95 to -0.20)		-0.15 (-0.42 to 0.13)	
≥A-level	-1.20 (-1.50 to -0.89)		-0.29 (-0.47 to -0.10)	
Frequency of contact				
Daily	-0.59 (-0.80 to -0.38)	0.158	-0.48 (-0.62 to -0.33)	0.023
Less than daily	-1.11 (-1.45 to -0.77)		-0.18 (-0.39 to 0.03)	

Fully adjusted linear models included demographics, socioeconomic, lifestyle, and health covariates and weighted to account for differential sampling and participation probabilities. Analyses were stratified by subgroups, with general cognitive factor scores re-standardised within each subgroup. Interactions were assessed using Wald F-tests. P-values for interaction were FDR-corrected.

## 4 Discussion

In this population-based study, self- and informant-reports showed broadly similar proportions and patterns of ADL and IADL impairment, but concordance was modest. Informant-reported impairments were more strongly and consistently associated with poorer cognitive performance—particularly in executive function, memory, and general cognition—and incident dementia. Associations differed by item: both self- and informant-reported impairment with managing money were associated with poorer cognition, whereas informant-reported problems with navigation in familiar or indoor spaces showed strong associations with dementia risk. These associations were independent of socioeconomic, lifestyle, and health factors, and were stronger among individuals with intermediate education and when informants had higher education or daily contact.

The similar proportions of functional impairment across self- and informant-ratings were consistent with previous findings in samples comprising individuals with normal cognition, MCI, and dementia[20] IADL impairments were generally more common than ADL impairments, consistent with findings from North America and India[[42], [43], [44]] At the item level, dressing and bathing were the most common ADL limitations, while eating and toileting were less frequent, in line with evidence that bathing and dressing decline early, whereas eating and toileting are typically lost later[25,45] This sequence may reflect the strong influence of physical health, with tasks requiring lower extremity strength, such as bathing, declining before those relying on upper extremity function[46] In China, however, toileting is often the first ADL lost,[47] likely due to the physical demands of squat toilets, underscoring how living environments shape ADL patterns. By contrast, IADL impairments showed more consistent patterns across settings and age groups,[46,48] with cognitively demanding tasks such as shopping, remembering short lists, and household tasks being most common, they also found to be earliest emerging IADLs that preceding any ADL decline[25,45] Notably, several IADL items included only in the informant questionnaire (e.g., “recalling recent events” and “remembering short lists”) were endorsed by a large proportion of informants (>30%), suggesting that they may more directly capture subtle early cognitive changes.

We found that compared with self-reports, informant-reports were more strongly and consistently related to objective cognitive performance. This pattern aligns with previous evidence that self-awareness of functional decline varies across the cognitive continuum, with individuals either overstating difficulties or losing insight as cognitive impairment progresses[[14], [15], [16]] Such non-differential misclassification in self-reports may yield similar overall frequencies of impairment as informant reports but bias associations with cognition towards the null. Beyond differences related to reporting source, the overall definitions of self- and informant-reported IADL impairment using different thresholds, with a higher bar for informant-reported impairment (BDRS Part 1 ≥2) than for self-report (any IADL difficulty). However, sensitivity analyses using a lower informant-reported threshold (BDRS Part 1 ≥1.5) yielded associations with cognitive function that were comparable to self-report, while associations with dementia remained stronger. Moreover, at the item level, informant ratings were dichotomised such that even mild functional difficulties were classified as impairment. Together, these findings indicate that differences in thresholds alone do not fully account for the observed pattern. Previous studies have likewise shown that informant-reported functional impairment correlates more strongly with cognitive impairment and neuropathological abnormalities across clinical groups (normal cognition, MCI, or dementia),[20] as well as progression from MCI to dementia[16] Our findings extend this evidence to incident dementia identified from medical records, an approach less prone to attrition bias.

In line with earlier research, memory and executive function were the cognitive domains most strongly associated with impairment with IADL[8,49,50] A prior meta-analysis reported stronger links with executive function than memory, but did not distinguish between self- and informant-ratings[51] By contrast, we found self-reported impairment to be more strongly related to executive function than with memory, whereas informant-reports showed stronger associations with memory than executive function, even for comparable items such as managing money. Because episodic memory might be a stronger predictor of dementia than executive function,[52] these findings highlight the greater value of informant-reported functional impairment for dementia risk assessment.

Specific functional items provided more informative signals. Self- and informant-ratings of managing money were closely correlated and showed the strongest associations with cognition, echoing findings across diverse settings and instruments[53] Financial management is cognitively complex, engaging both executive and memory processes;[54] importantly, its impairment can emerge up to a decade before dementia diagnosis[29] In our analyses, memory-related informant-reported IADL items, such as recalling recent events and remembering short lists, also showed strong associations with dementia, with difficulties finding one’s way in familiar environments standing out as the strongest. Similar items in other measures (e.g. “gets lost in community” in Community Screening Instrument for Dementia) have likewise shown the strongest associations with global cognition[53] This aligns with evidence linking spatial navigation deficits to structural and functional changes in neural networks affected early in dementia,[55] and to subsequent risk of MCI[56] By contrast, self-reported wayfinding with a map—which relies less on memory for familiar contexts—was only weakly associated with cognition and dementia, underscoring the importance of memory-based functional items for dementia algorithms.

For both informant-reported ADLs and IADLs, associations were strongest among those with intermediate education compared with the highest and lowest levels. Similar findings were reported across the US, England, and India,[57] where participants with some education and lower cognitive function showed closer alignment between informant reports and objective cognition than those with either the least education or the highest cognitive function. This may reflect greater functional reserve in more educated individuals, while informant ratings tend to be less accurate for those with low education[23] Previous studies have also shown that informants with frequent contact or close relationships provide more accurate ratings of functional ability than those with less contact[23,24] Furthermore, informants with higher education tend to report more IADL impairments, likely reflecting greater awareness of early symptoms of MCI and dementia[58,59] Our findings extend this evidence by showing that these informant characteristics may shape the degree to which reported impairments reflect underlying cognitive function.

Strengths of this study include being the first to compare self- and informant-reported functional measures in a population-based setting, the extension of exposures to individual items, and outcomes to domain-specific cognitive function derived from comprehensive cognitive batteries, as well as dementia identified through combined self-report and medical records. Several limitations should also be noted. Longitudinal ageing studies such as ELSA are subject to attrition[32] The HCAP subsample oversampled individuals with lower cognitive function, but participation remained biased toward individuals with higher education, better cognition, and fewer functional impairments[60] Although survey weights were used to adjust for sampling and participation differences, the findings may not be fully generalisable to the wider ageing population. The relatively low prevalence of cognitive impairment in this population-based sample, together with the absence of clinically adjudicated cognitive staging (e.g., cognitively normal, MCI, or dementia), precluded examination of associations across detailed stages of cognitive decline. Future studies designed to include sufficient representation across cognitive stages will be needed to determine whether the stronger associations observed for informant-reported functional impairment extend across the full spectrum from normal cognition to early and late mild cognitive impairment. Because self- and informant-reported functional impairment were assessed using different instruments, differences in their associations with cognitive performance and dementia may arise from the reporting source, the constructs assessed, item wording, response formats, or impairment thresholds. To help disentangle these factors, future studies could refine currently generic self-reported measures by incorporating memory-related items and more graded response options used in informant assessments, and evaluate whether such adaptations improve sensitivity to cognitive impairment and dementia risk. Domain-specific cognitive outcomes were restricted to language, memory, and executive function; other domains such as visuospatial ability were not included because of insufficient items and non-normal distributions. Although missingness in individual cognitive tests was addressed using established imputation procedures when deriving domain-specific factor scores, results may remain sensitive to departures from the missing-at-random assumption. The cohort was predominantly White; therefore, the findings may not be generalised to non-White individuals. Future research should include more ethnically diverse samples, as prior evidence indicates that cultural and ethnic differences influence both dementia prevalence and reporting of functional impairment. Lower impairment has been reported by African American informants[58] Cross-country comparisons further suggest weaker associations between functional impairment and global cognition in India than in the US and England[53] Finally, we lacked data on other factors that may influence the accuracy of informant ratings, such as caregiver burden and depression[61]

To conclude, this study highlights that informant-reported impairments with IADLs—particularly those reflecting complex abilities, episodic memory, and visuospatial memory—may be strong indicators of cognitive impairment–related functional loss, especially among individuals with intermediate education and when informants have higher education or daily contact. As these findings are derived from a population-based sample subject to attrition, validation in cohorts with different sampling and participation profiles is needed. Tailoring functional scales to incorporate these insights may improve the accuracy, timeliness, and scalability of dementia screening.

## Declaration of Generative AI and AI-assisted technologies in the writing process

I have not used any AI at all.

## Data sharing statement

ELSA-HCAP data are available to researchers after registration with the UK data service at https://datacatalogue.ukdataservice.ac.uk/studies/study/8502.

## Consent statement

The ELSA-HCAP study was conducted in accordance with the Declaration of Helsinki, and ethical approval and experimental protocols were granted by the South Central—Berkshire Research Ethics Committee (REC Ref: 17/SC/0254). All participants gave their informed consent to take part in the study.

## CRediT authorship contribution statement

**Yaqing Gao:** Writing – original draft, Visualization, Methodology, Formal analysis, Conceptualization. **Paola Zaninotto:** Writing – review & editing, Supervision. **Andrew Steptoe:** Writing – review & editing, Supervision, Methodology, Funding acquisition.

## Declaration of competing interest

The authors declare that they have no known competing financial interests or personal relationships that could have appeared to influence the work reported in this paper.

## Appendix Supplementary materials

Image, application 1
