# Supplementary material for: Associations of self- and informant-reported functional impairment with cognitive performance and incident dementia
Source: J Prev Alzheimers Dis. 2026 Jan 17;13(3):100482. doi: 10.1016/j.tjpad.2026.100482 (PMC12854031; doi:10.1016/j.tjpad.2026.100482)
Supplement: Supplementary file 1 [file mmc1.docx]

**Table S1**. Cognitive test items, domains, descriptions, and missingness in ELSA-HCAP

| Test | Item | Domain | Brief description | Missing proportion |
| --- | --- | --- | --- | --- |
| MMSE | Orientation to time | Orientation | Name month, year, day, season, date | 15/1273 (1.2%) |
| MMSE | Orientation to place | Orientation | Identify country, county, city, street, building | 1/1273 (0.1%) |
| MMSE | Object naming | Language | Name common objects (e.g., watch, pencil) | 1/1273 (0.1%) |
| MMSE | Command following | Language | Read and follow a written command (“close eyes”) | 14/1273 (1.1%) |
| MMSE | Phrase repetition | Language | Repeat a spoken phrase | 8/1273 (0.6%) |
| MMSE | Sentence writing | Language | Write a complete sentence | 44/1273 (3.5%) |
| MMSE | Serial 7 subtraction | Executive Functioning | Subtract 7s serially from 100 | 97/1273 (7.6%) |
| MMSE | Recall | Memory | Recall 3 words immediately | 5/1273 (0.4%) |
| MMSE | Recall | Memory | Recall 3 words after delay | 8/1273 (0.6%) |
| HRS-TICS | Vocabulary & factual knowledge | Language | Identify words (e.g., scissors, cactus) and name UK Prime Minister | 8/1273 (0.6%) |
| CERAD Word List | Immediate recall | Memory | Recall 10 words across three trials | 10/1273 (0.8%) |
| CERAD Word List | Delayed recall | Memory | Recall the 10 words after a delay | 9/1273 (0.7%) |
| CERAD Word List | Recognition | Memory | Recognize 10 original words among distractors | 12/1273 (0.9%) |
| CERAD Constructional Praxis | Immediate drawing | Visuospatial | Copy geometric figures (circle, cube, diamond, rectangles) | 68/1273 (5.3%) |
| CERAD Constructional Praxis | Delayed recall drawing | Memory | Redraw shapes from memory | 7/1273 (0.5%) |
| Animal Naming | Animal retrieval fluency | Language | Name as many animals as possible in 60s | 3/1273 (0.2%) |
| WMS-IV | Brave Man story – immediate | Memory | Recall story details immediately | 9/1273 (0.7%) |
| WMS-IV | Brave Man story – delayed | Memory | Recall story details after delay | 56/1273 (4.4%) |
| WMS-IV | Robbery story – immediate | Memory | Recall story details immediately | 20/1273 (1.6%) |
| WMS-IV | Robbery story – delayed | Memory | Recall story details after delay | 63/1273 (4.9%) |
| WMS-IV | Robbery story – recognition | Memory | Recognize true/false statements about story | 51/1273 (4.0%) |
| Ps & Ws Letter Cancellation | Letter cancellation | Executive Functioning | Cross out Ps and Ws on a letter grid in 60s | 87/1273 (6.8%) |
| Backward Counting | Backward counting | Executive Functioning | Count backwards from 100 for 30s | 20/1273 (1.6%) |
| SDMT | Symbol-digit substitution | Executive Functioning | Pair symbols with digits under time pressure | 77/1273 (6.0%) |
| Number Series | Numeric reasoning | Executive Functioning | Solve incomplete numeric sequences | 120/1273 (9.4%) |
| Raven's progressive matrices | Pattern reasoning | Executive Functioning | Identify missing shapes in abstract visual patterns | 15/1273 (1.2%) |
| Trail Making Test | Part A | Executive Functioning | Connect numbered circles in order (speed test) | 64/1273 (5.0%) |
| Trail Making Test | Part B | Executive Functioning | Alternate connecting numbers and letters | 235/1273 (18.5%) |
| CSI-D | Naming Items | Language | Object naming, usage, directions, pointing | 2/1273 (0.2%) |
| Other tasks | Paper-folding (3-step) | Language | Follow a three-step paper-folding instruction | 5/1273 (0.4%) |
| Other tasks | Interlocking shapes | Visuospatial | Draw overlapping/interlocking figures | 21/1273 (1.6%) |

MMSE, Mini-Mental State Examination; HRS-TICS, HRS Telephone Interview for Cognitive Status; CERAD, Consortium to Establish a Registry for Alzheimer's Disease; WMS-IV, Wechsler Memory Scale IV; SDMT, Symbol Digit Modalities Test; CSI-D, Community Screening Interview for Dementia. Proportions are unweighted.

**Figure S1**. Distribution of standardized cognitive factor scores in ELSA-HCAP.


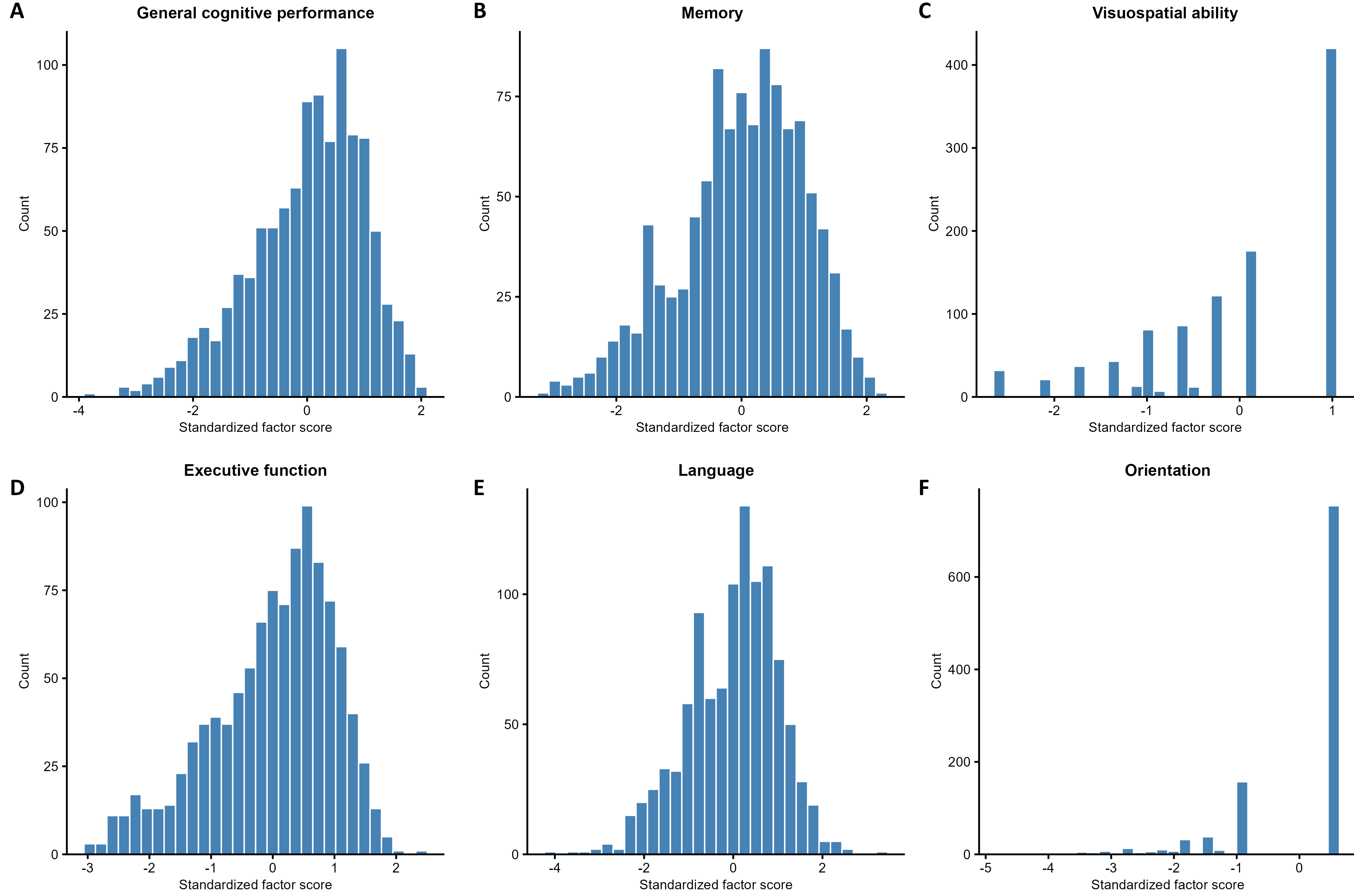


Histograms show the distribution of factor scores for (A) general cognitive performance, (B) memory, (C) visuospatial ability, (D) executive function, (E) language, and (F) orientation. Scores were estimated using confirmatory factor analysis, standardized to mean = 0 and variance = 1.

**Table S2**. ICD-10 codes used to define disease outcomes and covariates.

| Disease | ICD-10 code |
| --- | --- |
| Diabetes | E10, E11, E13 |
| Hypertension | I10, I11, I12, I13, I15 |
| Cardiovascular disease | I20, I21, I22, I23, I24, I25, I50, I60, I61, I63, I64 |
| Chronic lung disease | J41, J42, J43, J44 |
| Dementia ^a^ | A810, F00, F01, F02, F03, F051, F106, G30, G310, G311, G318, I673 |
| Arthritis | M15, M16, M17, M18, M19, M05, M06 |

ICD-10, International Classification of Diseases, Tenth Revision. List of diseases included as analytic outcomes or covariates, with the corresponding ICD-10 codes used to identify diagnoses from Hospital Episode Statistics (HES) records. Codes were specified at the three-character level (for example, F00), with all corresponding subcodes (for example, F00.0, F00.1) included by default.

^a^ A complete ICD-10 code list, including all three- and four-character subcodes: F00, F00.0, F00.1, F00.2, F00.9, G30, G30.0, G30.1, G30.8, G30.9, F01, F01.0, F01.1, F01.2, F01.3, F01.8, F01.9, I67.3, F02.0, G31.0, A81.0, F02.1, F02.2, F02.3, F02.4, F10.6, F02, F02.8, F03, F05.1, G31.1, G31.8

**Table S3**. Definitions, categorizations, and data sources for covariates used in the analyses

| Variable | Data source | Definition / categorization |
| --- | --- | --- |
| Age | HCAP | Interview year - birth year |
| Sex | HCAP |  |
| Living status | ELSA (wave 7/8) | Number of people in household: 1) Alone; 2) >1 Co-residing |
| Education | ELSA (wave 7/8, IFS derived) | Educational qualification (information merged from current and previous waves): 1) <O level; 2) O level; 3) ≥A level |
| Wealth | ELSA (wave 7/8, financial derived) | Benefit unit total net financial wealth (in quintiles). “Benefit unit” = single adult or couple living as married and dependent children. |
| Index of Multiple Deprivation | ELSA (wave 7/8) | Small-area socioeconomic measure (~1,500 residents) combining income, employment, education, health, crime, barriers to housing and services, and living environment. In quintiles. |
| Smoking status | ELSA (wave 7/8, IFS derived) | Cigarette smoking status: 1) Never; 2) Past/current (occasional, regular, unknown frequency ex-smoker, or current smoker) |
| Alcohol use | ELSA (wave 7/8) | Alcohol consumption in past 12 months: 1) ≥5 days/week (Almost every day; five or six days a week); 2) <5 days/week (Three or four days a week; once or twice a week; once or twice a month; once every couple of months; once or twice a year; not at all) |
| Informant sex | HCAP |  |
| Informant educational attainment | HCAP | Highest education level: 1) <O level (No qualifications; Trade apprenticeship); 2) O level (GCSE/O level grade); 3) ≥A level (A levels; Degree or degree equivalent) |
| Frequency of contact | HCAP | 1) Daily (Lives with respondent; Daily); 2) Less than daily (Once to several times/week; A couple times a month or less) |
| Diabetes | ELSA (wave 1-8), HCAP, HES | Self-reported diabetes/high blood sugar or HES diagnosis (ICD codes in Table S2) |
| Hypertension | ELSA (wave 1-8), HCAP, HES | Self-reported high blood pressure/hypertension or HES diagnosis (ICD codes in Table S2) |
| Cardiovascular disease | ELSA (wave 1-8), HCAP, HES | Self-reported angina, heart attack, heart failure, or stroke; or HES diagnosis (ICD codes in Table S2) |
| Chronic lung disease | ELSA (wave 1-8), HCAP, HES | Self-reported chronic lung disease (e.g., bronchitis, emphysema, chronic obstructive pulmonary disease) or HES diagnosis (ICD codes in Table S2) |
| Arthritis | ELSA (wave 1-8), HCAP, HES | Self-reported arthritis (including osteoarthritis or rheumatism) or HES diagnosis (ICD codes in Table S2) |
| Depressive symptoms | HCAP | CES-D items (yes/no): depressed, effort, restless sleep, happy, lonely, enjoyed life, sad, could not get going (past week) |

ELSA, English Longitudinal Study of Ageing; HCAP, Harmonised Cognitive Assessment Protocol; IFS, Institute for Fiscal Studies; HES, Hospital Episode Statistics; ICD, International Classification of Diseases; CES-D, Center for Epidemiologic Studies Depression Scale.

**Table S4**. Missing data for exposures and covariates

| Group | Test | Item / variable | Missing (n) | Missing (%) ^a^ |
| --- | --- | --- | --- | --- |
| Exposure | BDRS Part 1 | Remembering short lists | 65 | 6.2 |
| Exposure | BDRS Part 1 | Finding way around familiar streets | 57 | 5.4 |
| Exposure | BDRS Part 1 | Grasping situations or explanations | 39 | 3.7 |
| Exposure | BDRS Part 1 | Recalling recent events | 32 | 3.0 |
| Exposure | BDRS Part 1 | Coping with small sums of money | 31 | 3.0 |
| Exposure | BDRS Part 1 | Dwelling on the past | 31 | 3.0 |
| Exposure | BDRS Part 1 | Finding way indoors | 25 | 2.4 |
| Exposure | BDRS Part 1 | Performing household tasks | 23 | 2.2 |
| Exposure |  | Informant-reported IADL impairment ^b^ | 3 | 0.3 |
| Exposure | BDRS Part 2 | Using the toilet | 10 | 1.0 |
| Exposure | BDRS Part 2 | Dressing | 10 | 1.0 |
| Exposure | BDRS Part 2 | Eating | 3 | 0.3 |
| Exposure |  | Informant-reported ADL impairment ^c^ | 12 | 1.1 |
| Exposure | ADL | Eating | 2 | 0.2 |
| Exposure | ADL | Using the toilet | 2 | 0.2 |
| Exposure | ADL | Dressing | 2 | 0.2 |
| Exposure | ADL | Bathing | 2 | 0.2 |
| Exposure | ADL | Walking across a room | 2 | 0.2 |
| Exposure | ADL | Getting in or out of bed | 2 | 0.2 |
| Exposure | IADL | Finding way with a map | 2 | 0.2 |
| Exposure | IADL | Recognising danger | 2 | 0.2 |
| Exposure | IADL | Preparing a hot meal | 2 | 0.2 |
| Exposure | IADL | Shopping | 2 | 0.2 |
| Exposure | IADL | Making telephone calls | 2 | 0.2 |
| Exposure | IADL | Communication | 2 | 0.2 |
| Exposure | IADL | Taking medications | 2 | 0.2 |
| Exposure | IADL | Doing work around the house | 2 | 0.2 |
| Exposure | IADL | Managing money | 2 | 0.2 |
| Exposure |  | Self-reported ADL impairment ^d^ | 2 | 0.2 |
| Exposure |  | Self-reported IADL impairment ^e^ | 2 | 0.2 |
| Covariate |  | Informant educational attainment | 109 | 10.4 |
| Covariate |  | Alcohol use | 42 | 4.0 |
| Covariate |  | Wealth | 34 | 3.2 |
| Covariate |  | Index of Multiple Deprivation | 25 | 2.4 |
| Covariate |  | Frequency of contact | 21 | 2.0 |
| Covariate |  | Depressive symptoms | 10 | 1.0 |
| Covariate |  | Informant sex | 2 | 0.2 |
| Covariate |  | Education | 1 | 0.1 |
| Covariate |  | Age | 0 | 0.0 |
| Covariate |  | Sex | 0 | 0.0 |
| Covariate |  | Living status | 0 | 0.0 |
| Covariate |  | Smoking status | 0 | 0.0 |

^a^ Denominator = 1,050; ^b^ BDRS Part 1 score ≥2; ^c^ Difficulty with ≥1 items in BDRS Part 2; ^d^ Difficulty with ≥1 self-reported ADL; ^e^ Difficulty with ≥1 self-reported IADL; BDRS, Blessed Dementia Rating Scale; ADL, activities of daily living; IADL, instrumental activities of daily living. Proportions are unweighted.

**Table S5**. Associations of IADL impairment with cognition and dementia using different cut-offs

| Measure of IADL impairment | Proportion impaired (%) | Association with general cognitive function (β, 95% CI) | Association with incident dementia (HR, 95% CI) |
| --- | --- | --- | --- |
| BDRS Part 1 ≥ 2 | 19.8 | -0.36 (-0.48 to -0.25) | 5.00 (2.40 to 10.43) |
| BDRS Part 1 ≥ 1.5 | 29.6 | -0.28 (-0.39 to -0.18) | 3.82 (1.90 to 7.67) |
| Self-reported ≥ 1 item | 23.5 | -0.29 (-0.40 to -0.18) | 1.36 (0.67 to 2.79) |

Informant-reported instrumental activities of daily living (IADL) impairment was assessed using Part 1 of the Blessed Dementia Rating Scale (BDRS) with thresholds of ≥2 (primary definition) and ≥1.5 (less conservative definition). Self-reported IADL impairment was defined as difficulty with at least one IADL item. Associations with general cognitive function and incident dementia were examined using linear and Cox regression models, respectively. All models were adjusted for age, sex, socioeconomic, lifestyle, and health-related factors, with additional adjustment for informant characteristics when the exposure was informant-reported. Proportions and all regression models were weighted to account for differential sampling and participation probabilities. CI, confidence interval; HR, hazard ratio.

**Table S6**. Summary statistics for cognitive test performance in the final analytic sample (N = 1,050)

| Test | Item | Domain | Mean | SD | Min | p25 | p50 | p75 | Max | Missing proportion |
| --- | --- | --- | --- | --- | --- | --- | --- | --- | --- | --- |
| MMSE | Global cognitive function | Global | 26.7 | 3.0 | 14 | 25 | 27 | 29 | 30 | 110/1050 (10.5%) |
| HRS-TICS | Vocabulary & factual knowledge | Language | 2.7 | 0.6 | 1 | 3 | 3 | 3 | 3 | 5/1050 (0.5%) |
| CERAD Word List | Immediate recall | Memory | 17.4 | 5.6 | 0 | 14 | 18 | 21 | 30 | 7/1050 (0.7%) |
| CERAD Word List | Delayed recall | Memory | 5.0 | 2.7 | 0 | 3 | 5 | 7 | 10 | 7/1050 (0.7%) |
| CERAD Word List | Recognition | Memory | 8.8 | 1.9 | 0 | 8 | 10 | 10 | 10 | 10/1050 (1.0%) |
| CERAD Constructional Praxis | Immediate drawing | Visuospatial | 7.6 | 2.8 | 1 | 6 | 8 | 10 | 11 | 49/1050 (4.7%) |
| CERAD Constructional Praxis | Delayed recall drawing | Memory | 9.4 | 1.9 | 1 | 8 | 10 | 11 | 11 | 4/1050 (0.4%) |
| Animal Naming | Animal retrieval fluency | Language | 17.9 | 8.8 | 0 | 13 | 18 | 24 | 51 | 2/1050 (0.2%) |
| WMS-IV | Brave Man story – immediate | Memory | 3.9 | 1.6 | 0 | 3 | 4 | 5 | 6 | 5/1050 (0.5%) |
| WMS-IV | Brave Man story – delayed | Memory | 2.7 | 2.0 | 0 | 1 | 3 | 4 | 6 | 40/1050 (3.8%) |
| WMS-IV | Robbery story – immediate | Memory | 8.6 | 4.8 | 0 | 5 | 9 | 12 | 24 | 16/1050 (1.5%) |
| WMS-IV | Robbery story – delayed | Memory | 6.5 | 4.8 | 0 | 3 | 6 | 10 | 21 | 47/1050 (4.5%) |
| WMS-IV | Robbery story – recognition | Memory | 11.0 | 2.9 | 0 | 9 | 11 | 13 | 15 | 39/1050 (3.7%) |
| Ps & Ws Letter Cancellation | Letter cancellation | Executive Functioning | 15.9 | 5.3 | 0 | 13 | 16 | 19 | 36 | 57/1050 (5.4%) |
| Backward Counting | Backward counting | Executive Functioning | 30.2 | 11.6 | 0 | 23 | 31 | 38 | 74 | 15/1050 (1.4%) |
| SDMT | Symbol-digit substitution | Executive Functioning | 32.6 | 13.0 | 0 | 23 | 33 | 42 | 72 | 59/1050 (5.6%) |
| Number Series | Numeric reasoning | Executive Functioning | 526.7 | 34.4 | 409 | 514 | 529 | 547 | 584 | 88/1050 (8.4%) |
| Raven's progressive matrices | Pattern reasoning | Executive Functioning | 13.2 | 3.7 | 0 | 12 | 14 | 16 | 17 | 11/1050 (1.0%) |
| Trail Making Test | Part A | Executive Functioning | 59.3 | 41.2 | 4 | 36 | 48 | 67 | 300 | 45/1050 (4.3%) |
| Trail Making Test | Part B | Executive Functioning | 124.1 | 62.7 | 10 | 77.8 | 108 | 153 | 300 | 182/1050 (17.3%) |
| CSI-D | Naming Items | Language | 3.9 | 0.3 | 1 | 4 | 4 | 4 | 4 | 1/1050 (0.1%) |
| Other tasks | Paper-folding (3-step) | Language | 2.9 | 0.4 | 0 | 3 | 3 | 3 | 3 | 4/1050 (0.4%) |
| Other tasks | Interlocking shapes | Visuospatial | 0.9 | 0.3 | 0 | 1 | 1 | 1 | 1 | 14/1050 (1.3%) |

MMSE, Mini-Mental State Examination; HRS-TICS, HRS Telephone Interview for Cognitive Status; CERAD, Consortium to Establish a Registry for Alzheimer's Disease; WMS-IV, Wechsler Memory Scale IV; SDMT, Symbol Digit Modalities Test; CSI-D, Community Screening Interview for Dementia; SD, standard deviation; Min, minimum; p25, 25th percentile; p50, median; p75, 75th percentile; Max, maximum. Summary statistics are presented unweighted.

**Figure S2**. Correlation between self- and informant-reported ADL and IADL impairments at overall and item levels


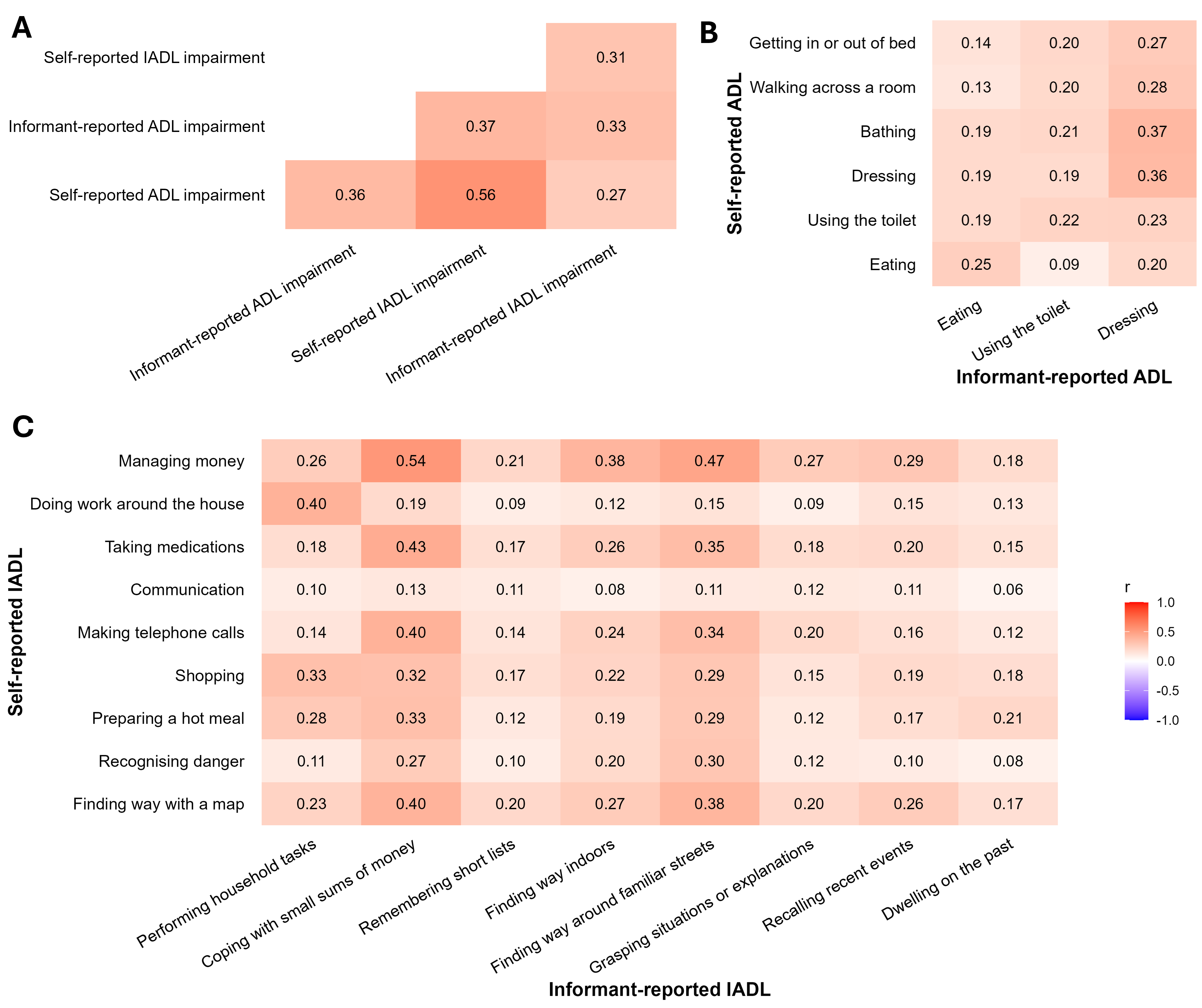


ADL, activities of daily living; IADL, instrumental activities of daily living. Heatmaps show pairwise Pearson correlation coefficients (r) between self- and informant-reported functional impairments. Colours indicate the strength of correlation, with darker red denoting stronger positive correlations. (A) Correlations between overall ADL and IADL impairment measures. (B) Item-level correlations for ADL impairments. (C) Item-level correlations for IADL impairments. All estimated correlation coefficients were weighted to account for differential sampling and participation probabilities.

**Table S7**. Associations between functional impairment and cognitive performance with sequential covariate adjustment

| Exposure | Outcome | Model 1 | Model 2 | Model 3 |
| --- | --- | --- | --- | --- |
| Self-reported ADL impairment | Executive function | -0.42 (-0.54 to -0.30) | -0.31 (-0.43 to -0.19) | -0.23 (-0.35 to -0.11) |
| Self-reported ADL impairment | Language | -0.21 (-0.35 to -0.08) | -0.12 (-0.25 to 0.02) | -0.06 (-0.20 to 0.08) |
| Self-reported ADL impairment | Memory | -0.11 (-0.24 to 0.02) | -0.02 (-0.15 to 0.11) | 0.08 (-0.06 to 0.21) |
| Self-reported ADL impairment | General cognition | -0.34 (-0.46 to -0.22) | -0.22 (-0.33 to -0.10) | -0.12 (-0.23 to 0.00) |
| Informant-reported ADL impairment | Executive function | -0.58 (-0.69 to -0.46) | -0.42 (-0.54 to -0.30) | -0.33 (-0.45 to -0.21) |
| Informant-reported ADL impairment | Language | -0.44 (-0.57 to -0.31) | -0.27 (-0.41 to -0.13) | -0.19 (-0.34 to -0.04) |
| Informant-reported ADL impairment | Memory | -0.63 (-0.75 to -0.51) | -0.53 (-0.67 to -0.40) | -0.46 (-0.60 to -0.32) |
| Informant-reported ADL impairment | General cognition | -0.64 (-0.75 to -0.52) | -0.46 (-0.58 to -0.34) | -0.36 (-0.48 to -0.25) |
| Self-reported IADL impairment | Executive function | -0.91 (-1.07 to -0.75) | -0.78 (-0.96 to -0.60) | -0.71 (-0.89 to -0.53) |
| Self-reported IADL impairment | Language | -0.73 (-0.91 to -0.54) | -0.70 (-0.91 to -0.49) | -0.59 (-0.80 to -0.38) |
| Self-reported IADL impairment | Memory | -0.55 (-0.72 to -0.37) | -0.54 (-0.74 to -0.33) | -0.46 (-0.66 to -0.25) |
| Self-reported IADL impairment | General cognition | -0.86 (-1.02 to -0.70) | -0.76 (-0.93 to -0.59) | -0.66 (-0.83 to -0.49) |
| Informant-reported IADL impairment | Executive function | -0.54 (-0.65 to -0.43) | -0.44 (-0.55 to -0.33) | -0.36 (-0.47 to -0.24) |
| Informant-reported IADL impairment | Language | -0.37 (-0.50 to -0.25) | -0.29 (-0.41 to -0.16) | -0.23 (-0.36 to -0.10) |
| Informant-reported IADL impairment | Memory | -0.31 (-0.43 to -0.19) | -0.25 (-0.37 to -0.13) | -0.16 (-0.29 to -0.03) |
| Informant-reported IADL impairment | General cognition | -0.50 (-0.61 to -0.39) | -0.39 (-0.50 to -0.29) | -0.29 (-0.40 to -0.18) |

ADL, activities of daily living; IADL, instrumental activities of daily living. Linear regression was used to examine associations between overall self- or informant-reported ADL/IADL impairment and cognitive performance. Model 1 adjusted for age and sex; Model 2 additionally adjusted for socioeconomic and lifestyle factors (and for informant characteristics when the exposure was informant-reported); Model 3 further adjusted for health-related variables. All models were weighted to account for differential sampling and participation probabilities. Results are presented as β coefficients with 95% confidence intervals.

**Table S8**. Associations between functional impairment and incident dementia with sequential covariate adjustment

| Exposure | Model 1 | Model 2 | Model 3 |
| --- | --- | --- | --- |
| Self-reported ADL impairment | 1.57 (0.89 to 2.76) | 1.45 (0.78 to 2.72) | 1.19 (0.54 to 2.63) |
| Informant-reported ADL impairment | 4.08 (2.25 to 7.38) | 4.61 (2.10 to 10.10) | 3.13 (1.23 to 7.96) |
| Self-reported IADL impairment | 1.77 (1.03 to 3.04) | 1.68 (0.91 to 3.11) | 1.36 (0.67 to 2.79) |
| Informant-reported IADL impairment | 4.29 (2.53 to 7.26) | 5.87 (2.99 to 11.55) | 5.00 (2.40 to 10.43) |

ADL, activities of daily living; IADL, instrumental activities of daily living. Cox regression was used to examine associations between overall self- or informant-reported ADL/IADL impairment and incident dementia. Model 1 adjusted for age and sex; Model 2 additionally adjusted for socioeconomic and lifestyle factors (and for informant characteristics when the exposure was informant-reported); Model 3 further adjusted for health-related variables. All models were weighted to account for differential sampling and participation probabilities. Results are presented as hazard ratios with 95% confidence intervals.
